# NIA DIVISION OF AGING BIOLOGY

**DOI:** 10.1093/geroni/igae098.0332

**Published:** 2024-12-31

**Authors:** Stacy Carrington-Lawrence

**Affiliations:** National Institute on Aging, Baltimore, Maryland, United States

## Abstract

Dr. Carrington-Lawrence will discuss research priorities of the NIA Division of Aging Biology. Dr. Carrington-Lawrence will also be available for small group discussion.

